# Molecular landscape and therapeutic alterations in Asian soft‐tissue sarcoma patients

**DOI:** 10.1002/cam4.4725

**Published:** 2022-05-18

**Authors:** Meifu Gan, Chen Zhang, Liqing Qiu, Yue Wang, Hua Bao, Ruoying Yu, Rui Liu, Xue Wu, Yang Shao, Peifeng Hou, Zhenglei Fei

**Affiliations:** ^1^ Department of Pathology Taizhou Hospital of Zhejiang Province Affiliated to Wenzhou Medical University Linhai China; ^2^ Radiotherapy & Chemotherapy Department 2, HwaMei Hospital University of Chinese Academy of Science Ningbo China; ^3^ Hangzhou Cancer Institution, Affiliated Hangzhou Cancer Hospital Zhejiang University School of Medicine Hangzhou China; ^4^ Geneseeq Research Institute Nanjing Geneseeq Technology Inc Nanjing China; ^5^ School of Public Health Nanjing Medical University Nanjing China; ^6^ Department of Medical Oncology Fujian Medical University Union Hospital Fuzhou China; ^7^ Anorectal Surgery Ningbo Medical Center Lihuili Hospital NingBo China

**Keywords:** homologous recombination deficiency, microsatellite instability, next‐generation sequencing, soft‐tissue sarcoma, therapeutic gene alteration, tumor mutation burden

## Abstract

**Background:**

Soft‐tissue sarcoma (STS) is a rare solid malignant tumor with numerous histologic subtypes. Current studies on targeted therapy for STS are in preclinical and early‐phase trials. Genomic differences largely influence the prognosis of patients even with the same subtype. To investigate the genomic alterations (GAs) and the potential of targeted therapy in STS, we analyzed the genomic landscape, the therapeutic GAs, and biomarkers of immunotherapy in Chinese STS patients.

**Methods:**

Targeted sequencing covering 425 genes was performed, from which we obtained the results of tissue samples from 351 Chinese STS patients of all ages covering different histologic subtypes. Bioinformatics analysis of altered genes with nonsynonymous mutations, copy‐number variations, and gene fusions were performed. OncoKB therapeutic GAs and relevant biomarkers including TMB, MSI, and HRD were further examined for potential targeted therapy.

**Results:**

In total, 2743 GAs were identified in 330 genes with a median of 6 (1–38) per case. The top 11 frequently altered genes were: *TP53*, *MCL1*, *MDM2*, *CDK4*, *MYC*, *CDKN2A*, *GNAS*, *RB1*, *ATRX*, *CDKN2B*, and *FGFR1*. OncoKB defined therapeutic GAs were found in 23 genes in 43% of the patients. In general, 9.4% of the patients had high‐TMB, 2.8% had MSI, and 13.7% had HRD. A significant difference in the percentage of patients with OncoKB therapeutic GAs were observed between the most frequent two subtypes, leiomyosarcoma and liposarcoma. Altogether, 54% of the patients had the potential to respond to a targeted therapy.

**Conclusion:**

This study indicated the potential efficacy of targeted therapy on many STS patients, and also provided insight for novel precision therapy. The clinical efficacy of combining targeted therapy and immunotherapy can be further investigated.

## Graphical abstract

We report an overall landscape of genomic alterations as well as immunotherapy biomarkers in Chinese soft‐tissue sarcoma patients of all ages with different subtypes. we investigated the potential of targeted therapy in soft‐tissue sarcoma showing the proportion of patients with OncoKB defined therapeutic alterations, TMB high, MSI and HRD. This is the first comprehensive genomic landscape of soft‐tissue sarcoma in Asian patients supporting the potential efficacy of targeted therapy and immunotherapy. Our results provide insights for more options of personalized medicine to improve the prognosis for patients with such a rare cancer.

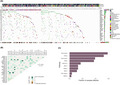

## 1 INTRODUCTION

Soft‐tissue sarcoma (STS) is a rare solid malignant tumor in adult, but it is one of the most common pediatric cancers. The incidence rate of STS in China is similar to western countries, which accounts for about 1% in adult cancers.^1^ STS has more than 50 histologic subtypes, originating from all over the body, which lead to various pathological and anatomical characteristics.^2^ The prognosis of STS patients is poor. In 2015, Asian‐Pacific region (STAR) study reported the median overall survival (OS) in adult patients with metastatic STS was 11.7 months and the 5‐year survival rate was less than 10% worldwide.^3^

For decades, due to the rarity and variety, the medical response in STS patients has been hardly improved with the generic first‐line conventional chemotherapeutic treatment which combines doxorubicin, ifosfamide, and other drugs, and the long‐term use usually causes a high risk of adverse events such as cardiomyopathy.^4^ Recent studies showed that apatinib, an antiangiogenesis tyrosine kinase inhibitor (TKI) specifically targeting *VEGFR‐2*, could prolong the progression‐free survival (PFS) for patients with advanced STS.^5^

With the development of next‐generation sequencing (NGS), targeted therapy can be more promising for various histologic STS subtypes assisting personalized treatments on different histologic subtypes of STS through genome profiling.^2^ In 2018, larotrectinib became the first approved targeted drug for both adults and pediatric patients with NTRK‐fusion associated sarcomas.^6^ While NTRK‐fusions usually exist in STS pediatric patients have *NTRK*‐fusions, this target is not common in adult patients.

Current studies on targeted therapy for STS are limited to preclinical and early‐phase trials.^2^ Biomarkers including tumor mutation burden (TMB), microsatellite instability (MSI), and homologous recombination deficiency (HRD) in STS patients have not been much reported so far. More comprehensive study on genome profiling to assist‐targeted therapies is urgently needed.

Poly (ADP‐ribose) polymerases (PARP) inhibitors have shown promising results in advanced STS, especially for Ewing sarcoma with *EWS‐FLI1* or *EWS‐ERG* genomic fusions, and PARP inhibitors have synergy effect with some chemotherapy such as trabectedin while the mechanism is not fully known.^7^ In this study, we analyzed the genomic landscape of Chinese STS patients with various subtypes and a wide range of ages to investigate the potential targetable genomic alterations. In addition, we investigated TMB, MSI, and HRD in the cohort.

## 2 METHODS

### 2.1 Patients and samples

In total, 388 Chinese STS patients were obtained from our clinical sequencing database. To keep the cohort size as large as possible, STS patients without available or reliable histologic subtypes were not excluded.

Sequences of tumor samples were profiled by GeneseeqPrime® NGS panel, which covered 425 cancer‐associated genes. The average sequencing coverage depth was 800X. To minimize DNA damaging effects and contaminations, samples did not pass in‐house quality control procedures were excluded. Tumor sample purity was calculated using ABSOLUTE.^8^ Samples which had less than 10% tumor contents were excluded. In the rest of samples, 11% had tumor contents between 10% and 20%, which might be considered having relatively low purity but had been kept in this study. The cohort had a mean purity of 48% and a range between 10% and 100%.”

Somatic SNVs and indels were called using Vardict, and were further filtered with the following criteria: (i) minimum ≥5 variant supporting reads and ≥1% variant allele frequency (VAF); (ii) present in <1% population frequency in the 1000 Genomes or ExAC database^9^, ^10^; (iii) present in an internally collected list of recurrent sequencing errors (≥3 variant reads and ≥1% VAF in at least 30 out of ~2000 normal samples) on the GeneseeqPrime® 425 panel. Finalized mutations were annotated using vcf2maf (v1.6.16, Cyriac Kandoth, https://github.com/mskcc/vcf2maf/releases/tag/v1.6.16). Gene‐level copy‐number variations (CNVs) were identified using FACETS.^11^ If the difference between the total copy number and ploidy is larger than or equal to 3, a CNV is defined as “gain,” and if it equals to 0, a CNV is defined as “loss.” Fusions were called by Delly with at least one splitting read and two discordant read‐pairs.^12^ All detected GAs were reviewed by our institution and reported to patients and physicians in the electronic medical record. Cell cancer fractions (CCFs) were calculated using ABSOLUTE.^8^

This study was approved by the ethical committee of each participating hospital and all patients provided written informed consent to participate. All samples were sequenced in a Clinical Laboratory Improvement Amendments (CLIA)‐ and College of American Pathologists (CAP)‐certified genomic testing facility (Nanjing Geneseeq Technology Inc.).

### 2.2 Molecular assays and therapeutic alterations

Nonsynonymous SNVs and indels in exons were selected and combined with CNVs and fusions as the total GAs, and 351 patients which had at least one GA were further analyzed.

The analysis of co‐occurrences and mutual exclusivity of genes with SNVs and indels were performed using maftools.^13^ The distribution of affected oncogenic signaling pathways was calculated according to the 10 canonical pathways profiled from The Cancer Genome Atlas.^14^

Therapeutic GAs were defined by OncoKB and classified into six therapeutic levels (FDA approved drugs, standard care, clinical evidence, biological evidence, and two resistant levels).^15^ TMB was defined as the total number of nonsynonymous mutations per mega‐base in coding regions. The panel TMB value was validated in an assay dataset and got CLIA/CAP accredited. TMB‐high cutoff was set as ≥10 mutations per Mb according to the widely accepted cutoff measured by FoundationOne panel and validated in a few studies.^16^ MSI testing was performed by using a customized analysis algorithm. The number of sequencing reads supporting each mononucleotide repeat length was calculated at each MSI site. The length distribution at the MSI site of the tumor sample was compared to a pool of normal samples. A site was considered instable if it showed significantly altered length distribution. In this study, samples were characterized as MSI if more than 40% of the sites evaluated by “GeneseeqONE MSI” showed instability.^17^ A comprehensive evaluation experiment was performed on GeneseeqONE MSI, and it achieved an accuracy of 95.6%, a sensitivity of 96.8%, and a specificity of 94.9% with a 40% cutoff when compared to Promega MSI Analysis System v1.2, and generated highly reproducible results within and between batches. HRD scores were determined by scarHRD, through calculating the genome‐wide allele‐specific copy‐number profile which was composed of loss of heterozygosity, telomeric allelic imbalance, and large‐scale state transitions.^18^ The HRD threshold (≥ 30) of the 425 panel was determined through the linear regression of HRD scores in 178 BRCA‐deficient samples (not in this cohort) which were sequenced using both the HRD panel and the 425 panel with a 95% sensitivity (Figure S1). The HRD panel which we use as the standard for HRD analysis detects about 10,000 SNPs, and the threshold in the HRD panel is close to the standard cutoff 42 in Myriad myChoice®. The linear regression shows that the HRD score cutoff 42 in the HRD panel corresponds to 30 in the 425 panel. Data mining, calculating, and visualization were performed using the R 4.0.3 (R Core Team, 2020) and packages including ggplot2, ComplexHeatmap, dplyr, and ggVennDiagram.^19^, ^20^

## 3 RESULTS

### 3.1 Molecular landscape

**FIGURE 1:**
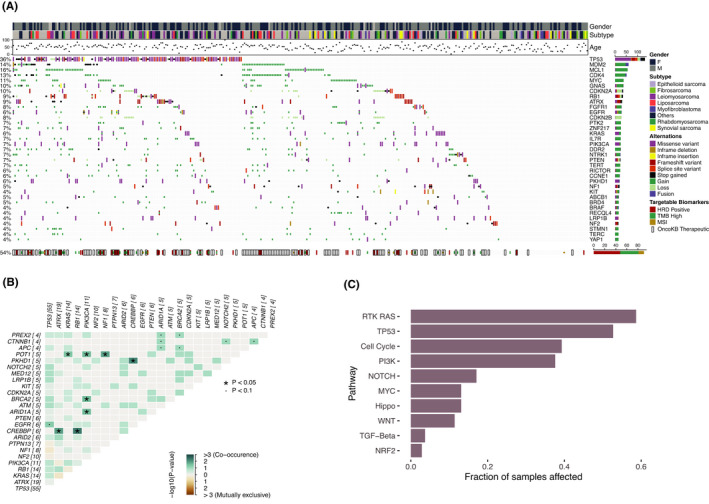
Landscape, co‐occurrence and related pathways of overall genetic alterations. (A) Landscape of prevalent genomic alterations with patient characteristics; (B) Co‐occurrence of genes with SNP and Indels; (C) Distribution of affected oncogenic signaling pathways

Out of 388 Chinese patients, 351 patients which got at least one GA were further analyzed. In these 351 patients, 45% were women and 55% were men. The median age was 54 years in a range from 2 to 99 years (Table 1). Patients without available subtype information were not excluded to keep the cohort size for GAs analysis in this study. Still, 28 histologic subtypes were found in the cohort, and the top seven relatively frequent subtypes were leiomyosarcoma (38), liposarcoma (27), fibrosarcoma (16), rhabdomyosarcoma (14), myofibroblastoma (14), epithelioid sarcoma (12), and synovial sarcoma (12) (Table 1 and Figure 1A Top). Consistent with previous research globally, leiomyosarcoma in the cohort was also significantly predominant in female (Female/Male ratio = 5.17).^21^, ^22^

**TABLE 1:** Patient characteristics

Sex	No. (Percentage)
Male	187 (53%)
Female	153 (44%)
Unknown	11 (3%)
Age at diagnose	
Range	2–99 years old
Median	54 years old
Unknown	68
Histologic subtype	Number of patients (percentage)
Leiomyosarcoma	38 (11%)
Liposarcoma	27 (8%)
Fibrosarcoma	16 (5%)
Rhabdomyosarcoma	14 (4%)
Myofibroblastoma	14 (4%)
Epithelioid. sarcoma	12 (3%)
Synovial. sarcoma	12 (3%)
Others	56 (16%)
Unknown	162 (46%)

A total of 2743 GAs in 330 genes were identified in tumor tissue samples, including 979 CNVs, 982 single nucleotide variations (SNVs), 182 indels, and 151 gene fusions (Table S1). The median number of GAs per patient was six in a range from 1 to 38. The top 10 genes were *TP53* (36%), *MCL1* (16%), *MDM2* (14%), *CDK4* (13%), *MYC* (11%), *CDKN2A* (10%), *GNAS* (10%), *RB1* (9%), *ATRX* (9%), *CDKN2B* (8%), and *FGFR1* (8%) (Figure 1A). Fusions were mostly occurred in *MDM2*, which corresponded to the results in a French sarcoma cohort with the other subtypes.^23^

Among the top three frequent genes with CNVs, the amplification of *MDM2* and *CDK4* were highly co‐occurred and they are in the proximity of positions on chromosome 12. Significantly co‐occurred oncogenic mutations (SNVs or indels) were shown in *CREBBP* with *ATRX*, *RB1,* and *PKHD1* separately, *POT1* with *KRAS*, *PIK3CA,* and *NF1* separately, and also *PIK3CA* with *ARID1A* and *BRCA2* separately (pair‐wise Fisher's Exact test, *p* < 0.05) (Figure 1B). No significant mutual exclusivity was found in the oncogenic mutations. Frequently affected oncogenic signaling pathways in the cohort were RTK/RAS (59%), TP53 (53%), Cell cycle (39%), and PI3K (38%) (Figure 1C).

### 3.2 Therapeutic genomic alterations

**FIGURE 2:**
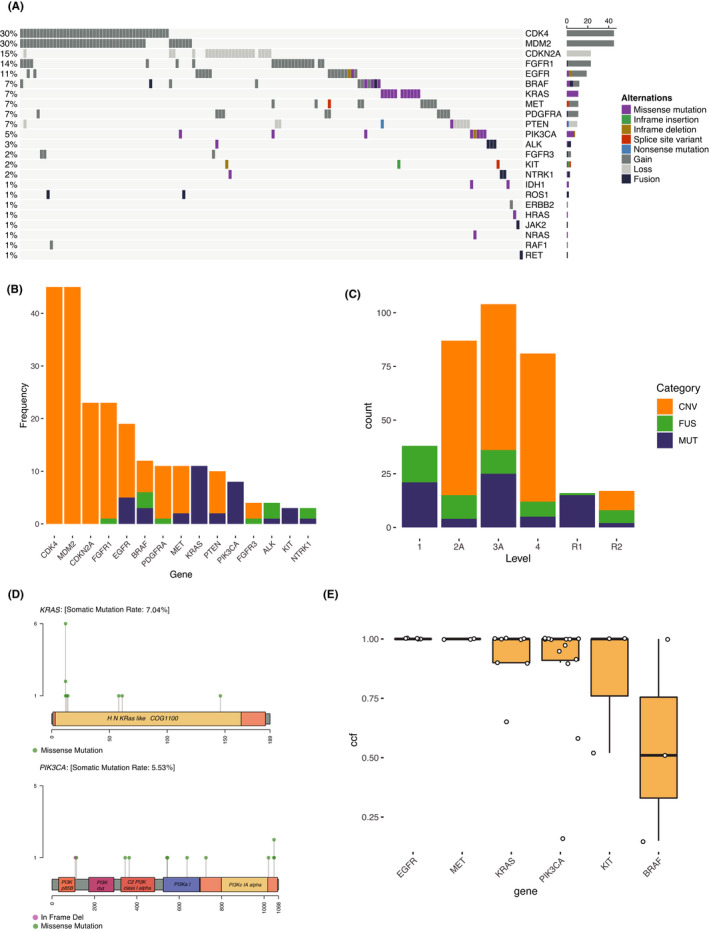
Landscape of OncoKB defined therapeutic genomic alterations. (A) Landscape of OncoKB defined therapeutic genomic alterations in the cohort; (B) Percentage of OncoKB defined therapeutic genomic alterations in top 15 genes (same as the vertical bar plot on the right side of Figure 2A but only categorized by mutation, CNV, and fusion); (C) Distribution of genomic alterations in OncoKB defined levels; (D) Locations of therapeutic SNVs and indels in *KRAS* and *PIK3CA*; E. Distribution of CCF of therapeutic mutations in genes with at least three such mutations

In 152 (43%) patients, at least one OncoKB defined therapeutic GA which could potentially assist‐targeted therapy was identified. A total of 242 therapeutic GAs were found in 23 genes **(**Figure 2A**)**. Top five genes with GAs which affected above 10% cohort were *CDK4* (30%), *MDM2* (30%), *CDKN2A* (15%), *FGFR1* (14%), and *EGFR* (11%). Therapeutic GAs included 187 CNVs, 40 mutations (SNVs and indels), and 15 gene fusions. Therapeutic CNVs were mostly found in *CDK4* (13%), *MDM2* (13%), *CDKN2A* (7%), and *FGFR1* (7%), and the therapeutic amplifications of *CDK4* and *MDM2* were mostly co‐occurred. Since samples could have multiple GAs in the same genes, the frequency of therapeutic GAs in top 15 genes were presented in Figure 2B. Although FDA defined therapeutic GAs were much more CNVs rather than mutations or gene fusions in the cohort, more mutations and gene fusions were targeted by current FDA approved medicines (Level 1) (Figure 2C). Therapeutic fusions found in this study except *PDGFRA* fusion (i.e., fusions in *FGFRR1*, *BRAF*, *FGFR3*, *ALK*, and *NTRK1*) were not shown in the French cohort.^23^ The two most frequent genes that harbored therapeutic SNVs and indels were *KRAS* (3%) and *PIK3CA* (2%), and the therapeutic mutation sites were shown in the lollipop‐style mutation diagrams (Figure 2D). In *KRAS*, therapeutic mutation hotspots were found in exon 2 (G12R, G12V, G12C, G12D, and G13D), exon 3 (Q61R), exon 4 (A146V).^24^ In *PIK3CA*, therapeutic hotspots were found in the helical (exon 9, E542K, and E545K) and in the catalytic domain (exon 20, H1047R).^25^ Five genes, *EGFR*, *MET*, *KRAS*, *PIK3CA*, *KIT,* and *BRAF*, had at least three therapeutic mutations, and all the median CCFs of the therapeutic mutations in the five genes were 100% except *BRAF* (Figure 2E). Since CCF is usually associated with the time of mutation occurrence, this result indicates that most of these therapeutic mutations happen at an early time.^26^ Also, OncoKB defined oncogenic mutations had CCFs significantly higher than CCFs of the other mutations in this study (Wilcox *p*‐value = 0.0064) as expected since oncogenic mutations tend to be early events during tumor evolution.

### 3.3 Potential patients for targeted therapy

**FIGURE 3:**
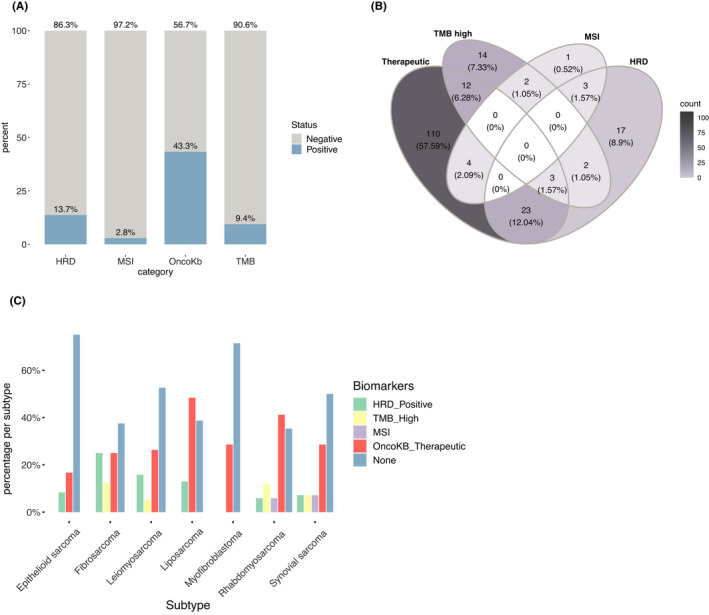
Distribution of targetable biomarkers. (A) Distribution of OncoKB, HRD, TMB, MSI in patients; (B) Relations of four targetable biomarkers; (C) Distribution of targetable biomarkers in seven frequent subtypes

Other predictive genomic biomarkers for immunotherapy include high‐TMB, HRD, and MSI.^27^, ^28^, ^29^ In the 351 patients, 14% had HRD, 9% had high‐TMB, and 3% had MSI (Figure 3A). Three biomarkers (therapeutic GAs, HRD, and high‐TMB) were found in three patients at the same time. Twenty‐three patients had both therapeutic GAs and HRD, and 12 patients had both therapeutic GAs and high‐TMB (Figure 3B). HRD was found in each of the seven relatively frequent subtypes except myofibroblastoma. None of the liposarcoma patients had high‐TMB (Figure 3C). Comparing the top two subtypes, liposarcoma patients had a significantly higher percentage of therapeutic GAs (Fisher's exact *p*‐value = 0.02165) (Table S2). However, adding HRD together, the targetable patients with liposarcoma did not increase, because liposarcoma patients with HRD also tend to have therapeutic GAs, while the potential of immunotherapy largely increase if considering all the biomarkers. Taking TMB, MSI, HRD, and therapeutic GAs altogether, 54% of the patients had the potential to respond to targeted therapy (Figure 1A Bottom and Table S3).

## 4 DISCUSSION

Several genomic analyses of STS have been published in recent years,^23^, ^30^, ^31^ but the situation in Chinese patients and relevant potential immunotherapy biomarkers in STS have not been fully investigated. One third of the frequent altered genes in this report were also observed in the French cohort ^23^, including *TP53*, *MDM2*, *CDK4*, *MYC,* and *CDKN2A*, among which *CDK4*, *MDM2,* and *CDKN2A* were the top three frequent GAs in this study, while the rest of frequent GAs, especially the amplification of *MCL1*, *GNAS,* and *FGFR1* might be more frequent specifically in Chinese STS patients or specific histologic subtypes. The co‐occurrence between *TP53* with *RB1* and *CDKN2A* were found in the previous research and suggested the simultaneous disruption of TP53 and cell cycle pathways.^23^ In our cohort, the co‐occurrence between *TP53* with *RB1* and *CDKN2A* existed but not significant. In addition, we observed the obvious co‐occurrence of the amplification of *MDM2* and *CDK4*, indicating the accompanying dysregulation of cell cycle pathways in STS tumorigenesis. *MDM2* and *CDK4* are known to be amplified in well‐differentiated and dedifferentiated liposarcoma.^2^ While gene fusions are commonly observed in STS (~20%), few OncoKB defined therapeutic fusions in adults were shown in previous research even in translocation‐related sarcomas.^23^ In this Chinese cohort, additional therapeutic fusions in *BRAF*, *ALK*, *NTRK1*, *FGFRR1,* and *FGFR3* were found.

Our results further confirmed that TMB and MSI were generally low in STS patients. If the MSI threshold was lowered to 30% as the most common cutoff in tumor research, patients with MSI would be improved from 2.8% to 6.6%. Previous research found that although *PD‐L1* was highly expressed in STS, patients did not clinically response to immune checkpoint inhibitors in general.^2^ One exception is alveolar soft part sarcoma (ASPS), which highly response to immune checkpoint inhibitors, but the mechanism has not been able to be explained by neither *PD‐L1*, TMB, nor MSI status so far.^2^

HRD in STS had not been investigated in previous research. In this study, a proportion of patients with HRD were found and potentially responsive to PARP inhibitors. The clinical effectiveness of the combination of PARP inhibitors and immune checkpoint inhibitors in STS are still under investigation and worth further studies.^32^

One major limitation of this study was that nearly half of the STS subtypes in the cohort were unknown and patients in rare subtypes were limited. Accurate and precise detection of GAs in rare subtypes is critical in targeted therapy. For example, while alterations in *ALK* tend to be gene fusions in STS, *ALK* alteration in rhabdomyosarcoma is mainly amplification, thus the investigated *ALK* inhibitor crizotinib is not effective for rhabdomyosarcoma.^33^ The molecular differences between histologic subtypes can be as huge as the diversity between solid tumors of different primary.^2^

Comprehensive genomic landscape brings novel insights in precision personalized STS treatment. In conclusion, this study provides indication on the potential efficacy of targeted therapy and immunotherapy in Chinese STS patients. OncoKB therapeutic GAs were observed in up to 41% of the cohort. Considering all the biomarkers together, 54% of the patients were potentially responsive to targeted therapy and immunotherapy. Clinical efficacy of combining targeted therapy and immunotherapy in STS can be further investigated.

## CONFLICT OF INTEREST

The authors Yue W, Hua B, Ruoying Y, Rui L, Xue W, and Yang S were employed by the company Nanjing Geneseeq Technology Inc. The remaining authors declare no potential conflict of interest.

## AUTHOR CONTRIBUTIONS

MG and CZ involved in conception and design. MG, CZ, PH, and ZF carried out provision of study material or patients. YW, HB, and RL involved in collection and/or assembly of data. YW, HB, RY, RL, XW, and YS analyzed and interpreted the data. ZF, PH, MG, CZ, and LQ wrote the manuscript. All authors read and approved the final manuscript.

## ETHICAL APPROVAL STATEMENT

This study was approved by the ethical committee of each participating hospital and all patients provided written informed consent to participate.

## Supporting information


Figure S1

Table S1

Table S2

Table S3

## Data Availability

Genetic sequencing data are available upon request, certain restrictions may apply.
