# Supplementary figures and images for: Molecular landscape and therapeutic alterations in Asian soft‐tissue sarcoma patients
Source: Cancer Med. 2022 May 18;11(21):4070–8. doi: 10.1002/cam4.4725 (PMC9636498; doi:10.1002/cam4.4725)

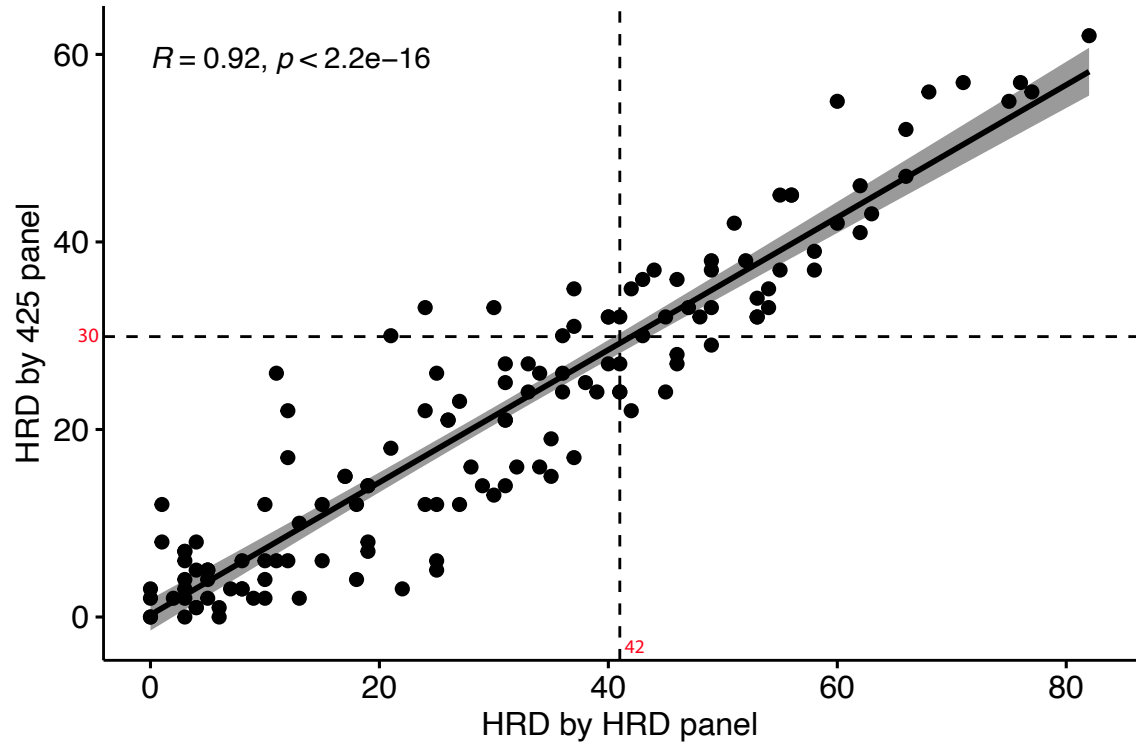

Supplement: Supplementary file 1 — Figure S1 [file CAM4-11-4070-s003.pdf]
